# The Causal Effect of Dietary Composition on the Risk of Breast Cancer: A Mendelian Randomization Study

**DOI:** 10.3390/nu15112586

**Published:** 2023-05-31

**Authors:** Hao Dong, Xiangyi Kong, Xiangyu Wang, Qiang Liu, Yi Fang, Jing Wang

**Affiliations:** National Cancer Center/National Clinical Research Center for Cancer/Cancer Hospital, Chinese Academy of Medical Sciences and Peking Union Medical College, Beijing 100021, China

**Keywords:** Mendelian randomization, breast cancer, protein, carbohydrate, fat, sugar

## Abstract

Breast cancer has become the most common malignancy among women, posing a severe health risk to women worldwide and creating a heavy social burden. Based on current observational studies, the dietary factor may have a causal relationship with breast cancer. Therefore, exploring how dietary composition affects breast cancer incidence will provide nutrition strategies for clinicians and women. We performed a two-sample Mendelian randomization (MR) analysis to find the causal effect of four kinds of relative macronutrient intake (protein, carbohydrate, sugar, and fat) on the risk of breast cancer and its subtypes [Luminal A, Luminal B, Luminal B HER2-negative, HER2-positive, Triple-negative, Estrogen receptor (ER) positive, and ER-negative breast cancer]. The Mendelian randomization pleiotropy residual sum and outlier (MR-PRESSO) test, MR-Egger intercept test, Cochran’s Q statistic, funnel plot, and leave-one-out (Loo) analysis were all used in a sensitivity analysis to test the robustness of MR. Genetically, a higher relative protein intake was found as a protective factor for Luminal A and overall breast cancer, which was inconsistent with recent findings. A higher relative sugar intake could genetically promote the risk of Luminal B and HER2-positive breast cancer. Conclusions: A higher protein proportion in diet genetically reduces the risk of breast cancer, while higher relative sugar intake does the opposite.

## 1. Introduction

Breast cancer in women has overtaken lung cancer as the most commonly diagnosed cancer worldwide. Approximately 2.3 million new cases of female breast cancer were diagnosed, which represents almost 12% of all cancer cases globally [1]. Furthermore, according to the GLOBOCAN Tomorrow Cancer prediction tool, the incidence of breast cancer is expected to increase by more than 46% by 2040, meaning breast cancer will bring a huge burden to society [2]. Meanwhile, compared to other malignancies, breast cancer leads to more disability-adjusted life years lost by women [3].

Dietary pattern has been testified as being associated with different diseases. Previous studies have also suggested that dietary factors could influence the risk of breast cancer in different ways [4,5,6]. For example, a higher dairy and total sugar intake could promote the risk of female breast cancer and other malignancies [7,8,9]. Furthermore, healthy dietary patterns, such as a higher vegetable, fruit, and soy product intake, can help reduce breast cancer risk [8,10]. Long-term observational studies have found an inverse association between breast cancer and the Mediterranean diet, characterized by a dietary pattern with abundant vegetables, fruits, fish, and olive oil [11,12]. As for the causal effect of dietary factors on breast cancer, the Global Cancer Update Programme has claimed that no causality can be inferred from current statistical correlations [6]. Therefore, figuring out the causal effect is a helpful measurement in conducting dietary intervention studies for women.

Randomized controlled trials (RCTs) are still the gold standard for identifying causal relationships [13]. Randomization enables studies to eliminate differences between subgroups to reduce bias. However, RCTs can neither eliminate all the confounders nor avoid the “reverse causation” that may influence the outcomes [14,15]. Mendelian randomization uses genetic variation as an instrumental variable (IV) to testify to the potential causal effects between exposures and outcomes [16]. It is important to note that the causality of genetic variation and traits is the foundation of MR. Because genetic variants are randomly assigned at conception, MR is not influenced by confounders that observational studies find difficult to avoid, making them a good proxy for cause-and-effect relationships [17].

**Figure 1:**
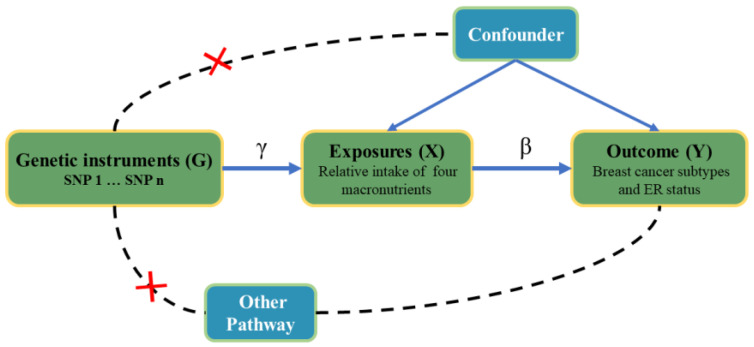
Study design overview and the hypothetical relationship between genetic variant, exposure, and outcome of the Mendelian randomization design. Allowed relationships between the variables are indicated by solid arrows, while dashed lines and red cross indicate relationships that are not permitted for G to qualify as a valid instrumental variable. The G–X and X–Y arrows are parameterized by γ and β, with the latter denoting the causal effect of X on Y.

Environmental and genetic factors can influence dietary habits [18,19]. In addition, the surrounding environment, including social and cultural factors, home and work environments, economic factors, and social support, can affect an individual’s sensitivity and preference for particular tastes, thereby influencing their dietary choices. In genetics, polymorphisms of some specific genes, such as fat mass and obesity-associated (FTO), melanocortin 4 receptor (MC4R), leptin receptor (LEPR), peroxisome proliferator-activated receptor-gamma (PPARG), and Adiponectin, have shown effects on weight gain, suppression of appetite, and oncogenesis [20,21,22,23,24,25]. The FTO gene displayed the most robust genetic correlation with polygenic obesity. FTO is commonly dysregulated and exerts significant effects on different categories of cancer. Meanwhile, FTO has the ability to stimulate cancer cell proliferation, enhance the self-renewal of cancer stem cells, and alter the immune and metabolic characteristics of cancer cells by eliminating the m6A modification from its target mRNAs and regulating their stability [24,26]. Based on the above facts, MR analysis can effectively analyze the causal relationship between dietary patterns and breast cancer risk from a genetic perspective. Several studies have already assessed the causal relationship between micronutrients (vitamin D, vitamin C, and vitamin E) and cancers [27,28,29], showing that dietary factors may contribute to the development of breast cancer. In comparison, there have been recent developments in the field of nutrition science indicating that the impact of diet on non-communicable diseases can be better explained by considering overall food consumption and dietary patterns rather than focusing solely on individual nutrients [30]. In this study, we have used the MR to investigate the potential causal relationship between the risk of breast cancer and four macronutrients in order to find robust genetic and phenotypic associations (Figure 1), giving some valuable suggestions for nutritional policies in the clinic.

## 2. Methods and Materials

### 2.1. Breast Cancer Data

We obtained the breast cancer risk genome-wide association studies (GWASs) summary data from the Breast Cancer Association Consortium (BCAC), which recruited over 100 groups with data on more than 200,000 individuals. Summary data on breast cancer risk came from these GWASs, including 133,384 breast cancer cases and 113,789 controls of European ancestry. In one GWAS, Zhang et al. used a novel two-stage polytomous regression method to characterize tumor heterogeneity by ER, progesterone receptor (PR), and human epidermal growth factor receptor 2 (HER2) status and tumor grade [31]. Moreover, the summary data for overall breast cancer and subtype-specific breast cancer (including Luminal A, Luminal B, Luminal B HER2-negative, HER2-positive, and Triple-negative breast cancer) risk were downloaded for free from the BCAC data resource [https://bcac.ccge.medschl.cam.ac.uk/bcacdata/oncoarray/oncoarray-and-combined-summary-result/gwas-summary-associations-breast-cancer-risk-2020/ (accessed on 13 February 2023)]. As for ER status, we used the summary data obtained from the GWAS conducted by Michailidou et al., including 21,468 ER-negative cases, 69,501 ER-positive cases, and 105,974 controls [32]. In addition, summary data with different ER statuses were also obtained from BCAC [https://bcac.ccge.medschl.cam.ac.uk/bcacdata/oncoarray/oncoarray-and-combined-summary-result/gwas-summary-results-breast-cancer-risk-2017/ (accessed on 13 February 2023)].

### 2.2. Relative Intake of Macronutrients Data

The IVs for the relative intake of the macronutrient data were obtained from the lead single-nucleotide polymorphisms (SNPs) of the GWAS conducted by Meddens et al. [33], which was performed on more than 235,000 individuals of European ancestry. Researchers got all participants’ dietary habits according to self-reports from the 24 h dietary recall (24HDR) questionnaire and food-frequency questionnaire (FFQ) [34,35]. In the discovery analyses, all the dietary information of the United Kingdom biobank (UKB) cohort was from the 24HDR. All participants with a verified email address were sent the questionnaire via email. They were requested to complete the questionnaire four times over a period of approximately one year (February 2011–April 2012) (https://biobank.ctsu.ox.ac.uk/crystal/refer.cgi?id=118240, accessed on 19 February 2023). In contrast, FFQ was used by all replication cohorts. The “macronutrient densities” are acquired by dividing the macronutrient intake by total energy intake. However, suppose the relative intake of macronutrients does not increase linearly with the total energy intake. In that case, the simple construction of macronutrient proportions may not be the optimal correction for total energy intake. As a result, the macronutrient intakes may need to be properly corrected for total energy intake, leading to residual correlations between macronutrient and total energy intake, which may vary by macronutrient. Meddens et al. [33] adopted corrected “macronutrient densities” ($\frac{energy\;from\;macronutrient}{total\;energy^{\beta }}$) with the correction factor β to measure the relative intake of macronutrient fat, protein, carbohydrates, and sugar.

### 2.3. Selection of Instrumental Variables

We used R (version 4.2.2) with the “TwoSampleMR” (version 0.5.6) package to perform the two-sample MR analysis. In this study, the summary data of exposure (relative intake of macronutrients) and outcome (breast cancer risk) came from different GWASs, which helped to reduce bias and improve precision. IVs are the only bridge to communicate exposure and outcome. Those SNPs regarded as IVs must satisfy the three following conditions: (i) they must exhibit strong associations with exposure (*p* < 5 × 10^−8^); (ii) they must only affect the outcome by exposure; (iii) they must have no relationship with the confounders [36]. According to the criteria mentioned above, IVs were clumped (*p* < 5 × 10^−8^, linkage disequilibrium (LD) r^2^ < 0.001, window size = 10,000 kb) from the lead SNP (Table S1), summarized by Meddens et al. [33,37]. Then, we harmonized the clumped data with the assistance of effect allele frequencies (EAF > 0.42), and palindromic variants would be deleted. Moreover, the outcome-related SNPs (*P* value of breast cancer risk < 5 × 10^−8^) were also removed from MR analysis. The variance of exposure [R^2^ = $\sum 2\times \beta^{2}\times EAF\times \left(1-EAF\right)$] was explained by the IVs of each macronutrient and calculated with β (genetic effect of each IV in exposure) and effect allele frequency (EAF). We calculated F statistics [$\left(\frac{N-K-1}{k}\right)$($\frac{R^{2}}{1-R^{2}}$)] with R^2^, sample size (N), and the number of instruments (K) (Table S1), considered as the index used to measure IV strength for MR analysis [38]. We used the online tool to calculate MR power (https://shiny.cnsgenomics.com/mRnd/, accessed on 13 February 2023). The power of MR analysis ranged from 5% to 94%, as shown in Table S2. The MR’s power values for relative protein intake between overall, Luminal A, and ER-positive breast cancer were 0.82, 0.20, and 0.94, respectively. As for sugar, the power of HER2-positive breast cancer was 0.83, while for the Luminal B breast cancer, no specific value was conducted using the online tool (for possible reasons, see the Discussion).

### 2.4. Mendelian Randomization Analysis

**Figure 2:**
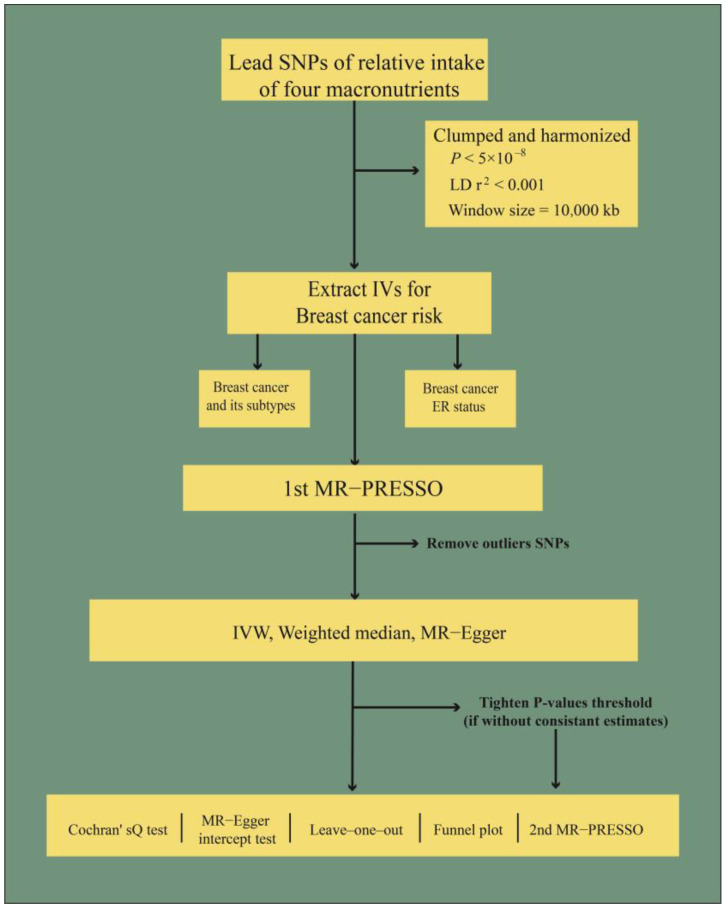
The flow chart of the MR analysis used in this study. Note: LD: linkage disequilibrium; ER: estrogen receptor; IV: instrumental variable; MR-PRESSO: Mendelian randomization pleiotropy residual sum and outlier test; IVW: inverse variance weighted.

To avoid the potential pleiotropic effect of the IVs, we performed different MR analysis methods to investigate the causal effect between the relative intake of four macronutrients and breast cancer risk in this study. Inverse variance weighted (IVW) estimates were considered as the primary methodology. IVW uses the Wald ratio from each variant to obtain the pooled causal effect. At the same time, there is the worry that it will underestimate the actual variation in the estimate, especially when the IV is weak [39]. Egger regression is used to detect bias from pleiotropy, and its slope coefficient estimates the causal effect. Egger regression can provide a consistent causal effect estimate even when IVs are invalid [40]. A weighted median estimator can combine data on multiple genetic variants into a single causal estimate and provide robust results, even with the number of invalid IVs being as high as 50% [41]. Moreover, the MR analysis flow chart is shown in Figure 2.

### 2.5. Sensitivity Analysis

Sensitivity analysis was performed to ensure the MR results’ robustness and reduce bias due to IV pleiotropy. Mendelian randomization pleiotropy residual sum and outlier (MR-PRESSO) test was used to test the horizontal pleiotropy (number of bootstrap replications = 10,000) and deleted the horizontal pleiotropic outliers to retest differences in the causal estimates of MR [42]. The intercept of Egger regression indicated the average pleiotropic effect across the IVs, manifesting overall directional pleiotropy (if *p* value < 0.05) [40]. Furthermore, Cochran’s Q statistic, funnel plot, and leave-one-out (LOO) analyses were all conducted to detect the presence of pleiotropy and assess the robustness of the results. Heterogeneity was considered when the *p* value of Cochran’s Q statistic was smaller than 0.05.

For the MR of the relative intake of macronutrients and risk of breast cancer, the putative causal effect would be considered if the *P* of MR met the Bonferroni correction (*p* < 0.0125, set as 0.05/4), and *p* < 0.05 was considered as nominally significant.

### 2.6. Sample Overlap

There were two partially overlapped studies [European Prospective Investigation into Cancer and Nutrition (EPIC) and Women’s Health Initiative (WHI)] included in the macronutrient composition and breast cancer risk GWAS. We considered the smallest sample size as an overlap in the same study. The EPIC and WHI sample overlap was 3.03% (7491/247,173) and 3.47% (8566/247,173) in the GWAS conducted by Zhang et al., respectively. The EPIC and WHI sample overlap was 3.08% (7057/228,951) and 3.74% (8566/228,951) in the GWAS conducted by Michailidou et al., respectively. Moreover, the proportion of sample overlap for the breast cancer risk GWAS conducted by Zhang et al. and Michailidou et al. was 6.50% (16,057/247,173) and 6.82% (15,623/228,951), respectively. The cohort information of the three studies and the overlapped samples are all shown in Table S3.

## 3. Results

### 3.1. Causal Effects

As for the risk of overall breast cancer, only relative intake of protein showed a strong genetically protective effect [Odds ratio (OR) = 0.64; 95% confidence interval (CI) = 0.45–0.89; *p* = 8.46 × 10^−3^, Table 1 and Figure S1a]. In the subtype analysis, the relative intake of protein also showed a genetically pronounced causal effect on lower incidences of Luminal A (OR = 0.50, 95% CI = 0.32–0.78, *p* = 2.21 × 10^−3^, Table 1 and Figure S1b) and ER-positive breast cancer (OR = 0.49, 95% CI = 0.32–0.74, *p* = 7.91 × 10^−4^, Table 1 and Figure S1c). On the contrary, relative intake of sugar would genetically increase the incidences of Luminal B (OR = 8.72, 95% CI = 2.31–32.88, *p* = 1.4 × 10^−3^, Table 1 and Figure S2a) and HER2-positive breast cancer (OR = 4.40, 95% CI = 1.44–13.43, *p* = 9.2 × 10^−3^, Table 1 and Figure S2b). Furthermore, the IVs for MR analysis were all shown in Figures S3d–f and S4c,d. Additionally, we observed that relative intake of carbohydrates can genetically promote the risk of breast cancer (OR = 1.61, 95% CI = 1.09–2.40, *p* = 1.79 × 10^−2^). However, the causal relationship cannot be testified via MR analysis because the *P* value failed to pass the Bonferroni correction (*p* = 1.25 × 10^−2^). Further sensitivity analyses have found no pleiotropy and heterogeneity for the above estimates. In the subgroup MR analysis of the relative intake of protein and HER2-enriched breast cancer, the IVW showed a protective trend in breast cancer incidence (OR = 0.31, 95% CI = 0.11–0.90, *p* = 3.13 × 10^−2^), while MR-Egger manifested an inconsistent nonsignificant estimate. Therefore, we tightened the *p* value of IVs to 5 × 10^−9^, and SNP rs445551 was removed. Further analyses showed inconsistent but nonsignificant estimates using three methods (Table S4). Finally, the MR results from MR-Egger and the weight median are shown in Table S5.

### 3.2. Sensitivity Analysis

To guarantee the robustness of causal estimates, we performed sensitivity analyses after each MR analysis (Table S5). Horizontal pleiotropy and outliers were found in the first MR-PRESSO test between the relative intake of protein and Luminal A as well as overall and ER-positive breast cancer (*p* < 1.00 × 10^−4^). When outliers (rs13146907, rs55872725, rs838133, as shown in Table S5) were removed, the pleiotropy was corrected. The same situation was also observed in the MR-PRESSO test between relative intake of sugar and Luminal B breast cancer. After outlier rs7012814 was deleted, no pleiotropy was detected. As for the HER2-positive breast cancer and relative intake of sugar, the first MR-PRESSO did not find any outliers. All the *p* values of Cochran’s Q and MR-Egger intercept tests were greater than 0.05 in the five significant estimates mentioned above. Egger intercepts did not detect any pleiotropy (Figures S1 and S2). Furthermore, the LOO analysis demonstrated that no SNP drove the findings (Figures S3d–f and S4c,d), and the funnel plots (Figures S3a–c and S4a,b) displayed a symmetrical distribution.

## 4. Discussion

In our study, we used MR analysis to explore the genetic causal relationship between the relative intake of four macronutrients and the risk of breast cancer. A higher relative intake of protein was found to be a protective factor against breast cancer. At the same time, a higher relative intake of sugar had shown a significant causal effect on breast cancer. As for the sensitivity analysis, MR-PRESSO was performed secondly, unless IVs were less than four or no outliers were detected (Table S5). Moreover, no IV pleiotropy and heterogeneity were found in the significant estimates after the correction of MR-PRESSO (Figures S3 and S4). Therefore, almost all the power values of the significant estimates were robust. As for the power calculation of sugar and HER2-positive breast cancer, there might be a limiting value instead of no value. Based on the original parameters, we have artificially set the ORs as 2.5, 3.5, 4.5, and 5.5, and the calculating power values as 0.37, 0.82, 1.00, and 1.00, respectively. There was a trend that when the OR value was increasing, the power was infinitely close to 1.00. Therefore, when the OR was equal to 8.72, the power value was robust enough to support the MR analysis.

Because the macronutrients are the primary energy source of the human body, there may be a possibility that the four macronutrients can influence the incidence of breast cancer through the pathway of obesity, which has been considered a risk factor for breast cancer [43]. Dennis et al. found that a low relative carbohydrate proportion and a high intake of fat genetically contribute to higher body mass index (BMI) and a higher waist circumference (WC); they found that there was no causal relationship between relative intake of protein and sugar and BMI and WC [44].

In this study, dietary structure has been considered a form of oncogenesis [45]. Macronutrients, such as fat, protein, and carbohydrate, provide almost all the energy and essential components required to satisfy human physiological activities. However, outcomes derived from observational studies of meat consumption and the incidence of breast cancer were still controversial [46,47,48]. Our results are inconsistent with most observational studies and meta-analyses that the red or processed meat was believed to induce breast cancer for the following reasons: (1) carcinogenic compounds, such as N-nitroso compounds, heterocyclic amines, and polycyclic aromatic hydrocarbons that can dose-dependently generate DNA adducts [49,50]; (2) inflammation and oxidative stress may arise from animal fat and iron-enriched red meat [51]. On the contrary, a recent meta-analysis suggested that a higher soy food intake would help decrease the risk of breast cancer [52]. Furthermore, a long-time large-scale observational study has also found that a higher soy intake could reduce the risk of breast cancer in postmenopausal women [53]. Soy is an essential source of vegetable protein. Its positive effect on breast cancer risk and breast cancer survival was attributed to isoflavone, a phytoestrogen, and selective estrogen receptor modulator [54]. Given the relationship between soy consumption and the risk of breast cancer mentioned above, it somewhat validated our MR finding that women would benefit from a higher dietary protein proportion.

A higher relative sugar intake is strongly associated with a higher risk of breast cancer in our MR analysis. Recently, a fasting-mimicking diet (FMD) has been testified to enhance the efficacy of standard cancer therapy. FMD, a dietary structure low in calories, sugars, and protein but relatively high in fat content, could strengthen endocrine therapeutics tamoxifen and fulvestrant by lowering circulating IGF1, insulin, and leptin by upregulating EGR1 and PTEN to inhibit the AKT–mTOR signal pathway [55]. Claudio et al. revealed that an FMD could lead to a consistent decrease in blood glucose and growth factor concentration, remodeling anticancer immunity via changes to peripheral and intratumor cellular components [56]. Notably, a higher dietary proportion of sugar could result in high glucose levels and promote high insulin levels in parallel, leading to cancer growth [57]. As a subtype of carbohydrates, sugar can be more rapidly absorbed and affect plasma glucose levels than other sources of carbohydrates, such as starch and dietary fiber. Potentially, it can lead to breast cancer via higher plasma glucose levels, which can, in turn, promote potential pathways. At the same time, the association between the risk of breast cancer and relative intake of carbohydrates was unclear in our study, which was consistent with current findings. In a prospectively observational study, the vegetable-based, low-carbohydrate diet habit was inversely associated with a reduced risk of ER-negative breast cancer [58]. Bahareh et al. have found that adherence to a low-carbohydrate diet may increase the risk of breast cancer in postmenopausal women. Long-term, large-cohort studies with a precise definition of a low-carbohydrate diet and scientific study design should be further performed.

We have failed to find the causal relationship between the risk of breast cancer and the relative intake of fat. At the same time, a large-scale, case–control study has revealed that replacing fat intake with carbohydrates of equal calories could lower the risk of breast cancer. Moreover, the same association was also shown for fat and protein [59]. Sabina et al. found that high total and saturated fat intakes were associated with a greater risk of hormone-receptor-positive breast cancer. Meanwhile, a high saturated fat intake was significantly associated with a greater risk of HER2-negative breast cancer [60]. Compared to protein and carbohydrates, fat can provide more energy per gram. Therefore, it means that a higher relative intake of fat is more likely to lead to obesity, which has already been considered a risk factor for breast cancer.

The strength of our study is that we, for the first time, investigated with the MR analysis the causal relationship between the relative intake of four macronutrients and the risk of breast cancer. In addition, the proportion of sample overlap in our study was small, guaranteeing the independence of exposure and outcome and making the estimates of MR more robust. Moreover, we used different methods to perform sensitivity analysis to detect outliers and correct potential pleiotropy and heterogeneity. On the other hand, most current diet-related research has concentrated on specific foods or ingredients, which may not offer comprehensive insights into the optimal diet for overall health. Instead, dietary patterns, which encompass a range of foods, nutrients, and beverages, can be valuable tools for assessing the overall impact of diet on health outcomes. The exposure information in our study is derived from large cohorts via dietary questionnaires, a process that reflects, to the greatest extent, the dietary structure of the participants in the cohorts. In addition, it indirectly describes the proportion of the four macronutrients in the dietary pattern using energy density.

Our study also had some limitations. Firstly, the original dietary information obtained from UK Biobank was collected via a 24HDR questionnaire. Moreover, participants were asked to recall as accurately as possible how many portions of each food item they had consumed the previous day. Compared with the FFQ used by other cohorts, feedback from the 24HDR may have more random variation since the FFQ can obtain dietary intakes for a standard day in the previous week or month. Secondly, the IV numbers for relative fat, protein, and sugar intake were too small, potentially weakening the strength of the MR analysis due to the convergence bias. Thirdly, only European ancestry was tested in our study, so it is unreasonable to interpret our findings in other populations. Finally, the findings in our study are based on the current background of GWASs, which have identified the causal relationship between macronutrient intake and the risk of breast cancer in genetics. This can provide some reference for the formulation of clinical nutrition strategies but still requires large-scale clinical trials for validation.

## 5. Conclusions

This study utilized the genetic method to investigate the causal relationship between the relative intake of macronutrients and the risk of breast cancer. We found that a higher relative intake of protein was inversely associated with the risk of Luminal A and overall breast cancer. On the contrary, a higher relative intake of sugar would promote incidences of Lumina B and HER2-positive breast cancer. However, what we have found was partially inconsistent with the current finding, so further validation needs to be performed via clinical trials.

## Figures and Tables

**Table 1:** Mendelian randomization results derived from IVW for macronutrient composition and breast cancer.

Exposure	Outcome (Breast Cancer)	Number of IVs	*p*	OR (95%CI)	Cochran’s Q Test	MR-Egger Intercept Test
**Relative intake of carbohydrate**	Overall	6	0.19	1.26 (0.89–1.80)	0.16	0.41
	Luminal A	5	**1.79 × 10^−2^**	1.61 (1.09–2.40)	0.44	0.76
	Luminal B	7	0.14	2.07 (0.78–5.50)	0.14	0.04
	Luminal B HER2-negative	8	0.87	0.95 (0.49–1.83)	0.34	0.64
	HER2-positive	8	0.89	1.10 (0.27–4.55)	0.07	0.91
	Triple-negative	8	0.61	0.83 (0.41–1.69)	0.25	0.51
	ER-negative	6	0.38	0.79 (0.46–1.34)	0.78	0.45
	ER-positive	7	0.85	1.04 (0.72–1.51)	0.26	0.33
**Relative intake of fat**	Overall	4	0.68	0.91 (0.57–1.44)	0.13	0.13
	Luminal A	4	0.54	0.86 (0.52–1.40)	0.16	0.17
	Luminal B	4	0.83	1.17 (0.29–4.80)	0.47	0.15
	Luminal B HER2-negative	4	0.52	0.74 (0.29–1.87)	0.13	0.15
	HER2-positive	4	0.16	0.51 (0.20–1.30)	0.76	0.94
	Triple-negative	4	0.15	1.50 (0.87–2.58)	0.98	0.90
	ER-negative	4	0.12	1.40 (0.92–2.12)	0.95	0.80
	ER-positive	4	0.67	0.89 (0.50–1.57)	0.26	0.11
**Relative intake of protein**	Overall	4	**8.46 × 10^−3^**	0.64 (0.45–0.89)	0.43	0.42
	Luminal A	4	**2.21 × 10^−3^**	0.50 (0.32–0.78)	1.00	0.96
	Luminal B	5	0.09	0.48 (0.21–1.13)	0.26	0.19
	Luminal B HER2-negative	6	0.99	1.00 (0.56–1.79)	0.56	0.32
	HER2-positive	6	0.06	0.31 (0.09–1.07)	0.16	0.42
	Triple-negative	5	0.87	0.94 (0.45–1.95)	0.79	0.95
	ER-negative	5	0.07	0.60 (0.35–1.04)	0.70	0.44
	ER-positive	4	**7.91 × 10^−4^**	0.49 (0.32–0.74)	0.54	0.34
**Relative intake of sugar**	Overall	3	0.27	1.36 (0.79–2.35)	0.17	0.31
	Luminal A	3	0.38	1.28 (0.74–2.18)	0.52	0.55
	Luminal B	4	**1.39 × 10^−3^**	8.72 (2.31–32.88)	0.06	1.00
	Luminal B HER2-negative	5	0.15	2.41 (0.72–8.03)	0.01	0.24
	HER2-positive	5	**9.19 × 10^−3^**	4.40 (1.44–13.43)	0.60	0.56
	Triple-negative	3	0.94	1.04 (0.37–2.89)	0.95	0.84
	ER-negative	4	0.90	0.96 (0.55–1.68)	0.73	0.62
	ER-positive	4	0.21	1.26 (0.88–1.82)	0.70	0.59

Note: IVW: inverse variance weighted; 95%CI: 95% confidence interval; IVs: instrumental variables; *p* = *p* value; outcome = risk of breast cancer.

## Data Availability

Publicly available GWAS data on breast cancer survival/susceptibility were obtained from https://bcac.ccge.medschl.cam.ac.uk/bcacdata (accessed on 14 February 2023). Genetic data on the relative intake of macronutrients were obtained from GWAS reported by Meddens et al. [33].

## Supplementary Materials

The following supporting information can be downloaded at: https://www.mdpi.com/article/10.3390/nu15112586/s1. Supplementary tables; Table S1: lead SNPs identified across diet composition GWAS; Table S2: the power of Mendelian randomization analysis in the study; Table S3: sample overlap of exposure and outcome genome-wide association studies; Table S4: details for Mendelian randomization studies with tightened instrument variables selection; Table S5: results of MR analysis and sensitivity analysis. Supplementary figures; Figure S1. scatter plots from genetically predicted protective effects assessed the relative intake of protein’s effect on breast cancer risk; Figure S2. scatter plots from genetically predicted protective effects assessed the relative intake of sugar on breast cancer risk; Figure S3. funnel plot and leave-one-out sensitivity analysis of MR estimate assessed the relative intake of protein and its effect on overall breast cancer risk; Figure S4. funnel plot and leave-one-out sensitivity analysis of MR estimate assessed the relative intake of sugar and its effect on overall breast cancer risk.
