# Supplementary material for: The Causal Effect of Dietary Composition on the Risk of Breast Cancer: A Mendelian Randomization Study
Source: Nutrients. 2023 May 31;15(11):2586. doi: 10.3390/nu15112586 (PMC10255061; doi:10.3390/nu15112586)
Supplement: Supplementary file 1 [file nutrients-15-02586-s001.zip › Supplementary Figures.pdf]

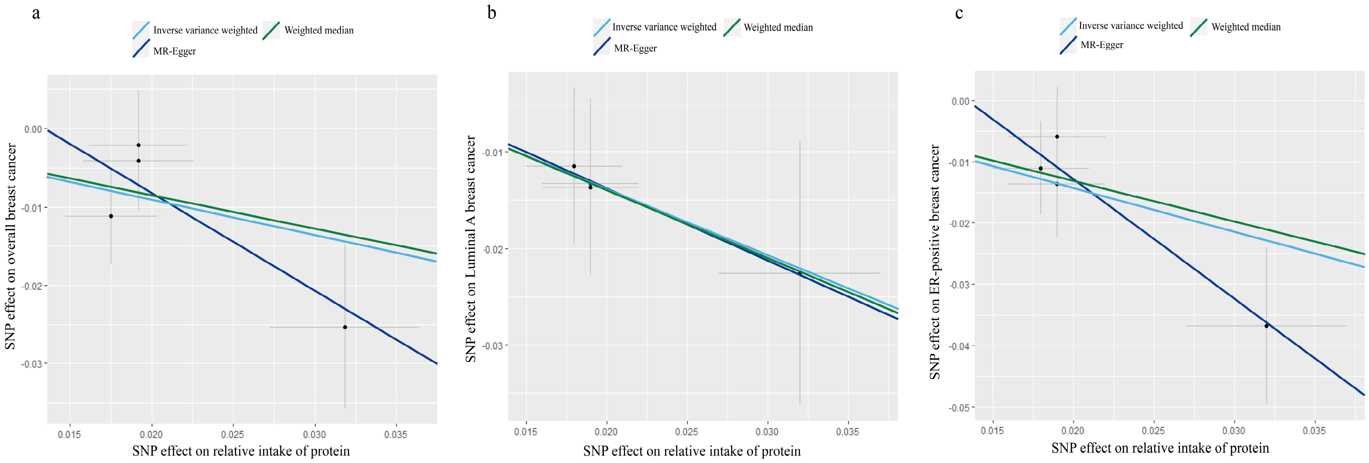

Figure S1. Scatter plots from genetically predicted protective effects assessed the relative intake of protein on breast cancer risk. (**a**) genetically predicted protective effects assessed the relative intake of protein on overall breast cancer risk; (**b**) genetically predicted protective effects assessed the relative intake of protein on Luminal A breast cancer risk; (**c**) genetically predicted protective effects assessed the relative intake of protein on ER-positive breast cancer risk.

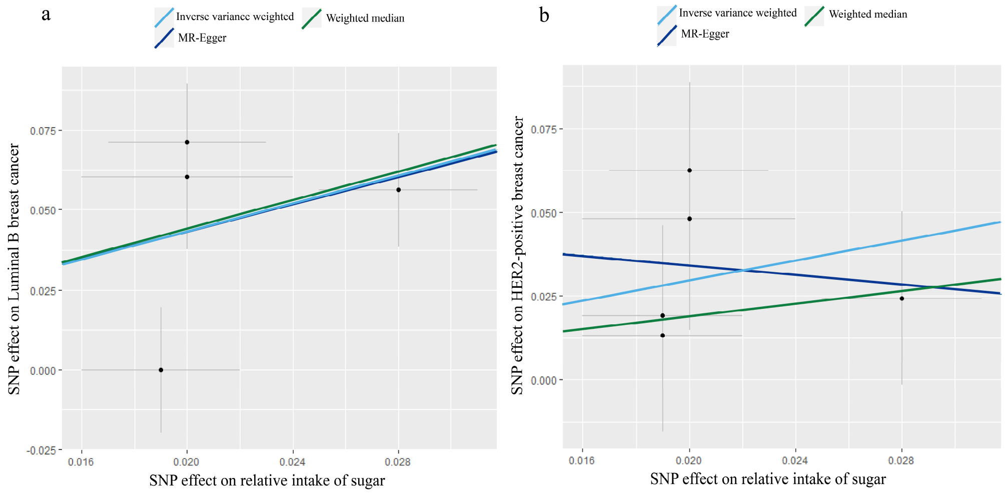

Figure S2. Scatter plots from genetically predicted protective effects assessed the relative intake of sugar on breast cancer risk. (**a**) genetically predicted protective effects

assessed the relative intake of sugar on Luminal B breast cancer risk; (**b**) genetically predicted protective effects assessed the relative intake of protein on HER2 positive breast cancer risk.

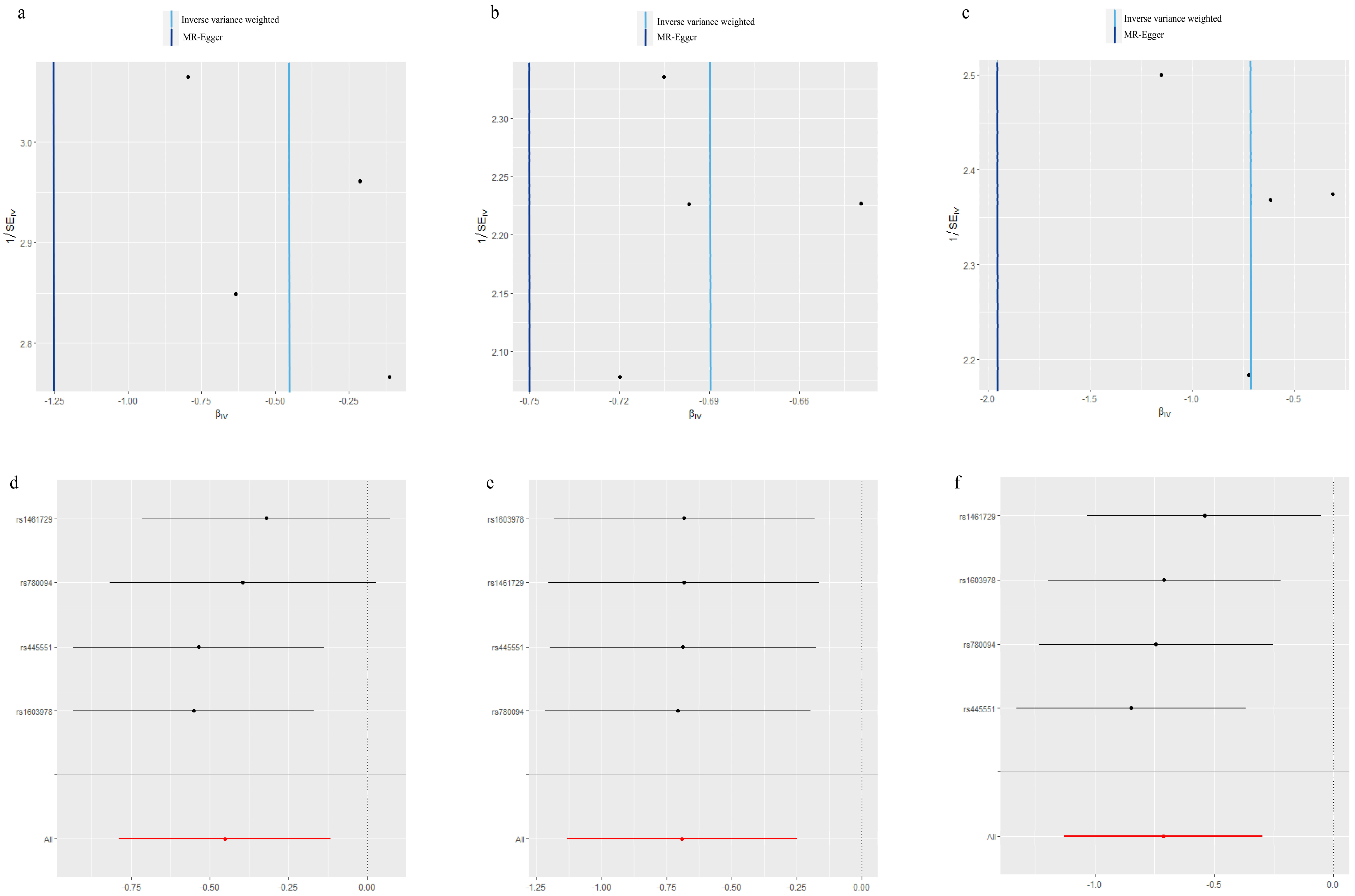

Figure S3. Funnel plot and Leave-one-out sensitivity analysis of MR estimate assessed the relative intake of protein on overall breast cancer risk. **(a)** funnel plot of MR estimate assessed the relative intake of protein on overall breast cancer risk; **(b)** funnel plot of MR estimate assessed the relative intake of protein on Luminal A breast cancer risk; **(c)** funnel plot of MR estimate assessed the relative intake of protein on ER-positive breast cancer risk; **(d)** Leave-one-out analysis of MR estimate assessed the relative intake of protein on overall breast cancer risk; **(e)** Leave-one-out analysis of MR estimate assessed the relative intake of protein on Luminal A breast cancer

risk; **(f)** Leave-one-out analysis of MR estimate assessed the relative intake of protein on ER-positive breast cancer risk.

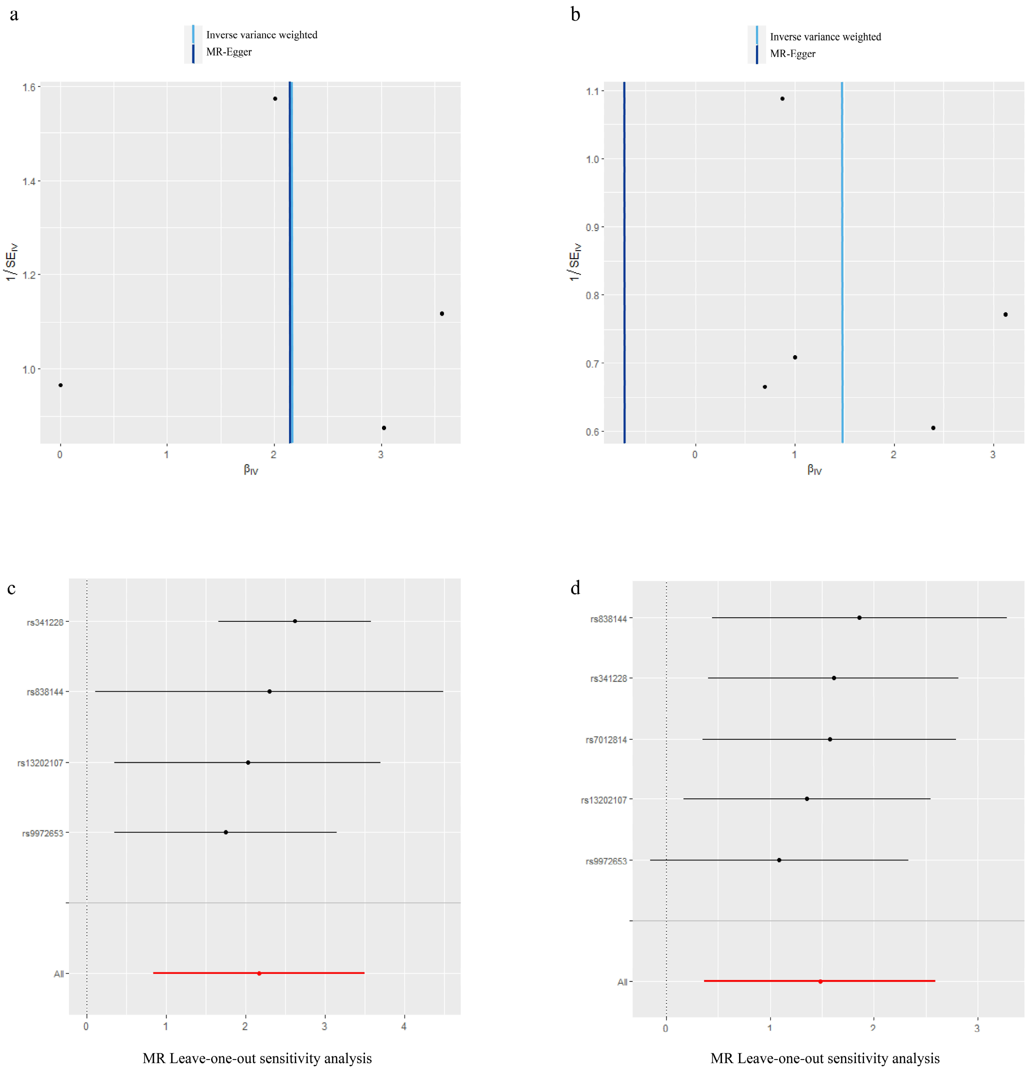

Figure S4. Funnel plot and Leave-one-out sensitivity analysis of MR estimate assessed the relative intake of sugar on overall breast cancer risk. **(a)** funnel plot of MR estimate assessed the relative intake of sugar on Luminal B breast cancer risk; **(b)** funnel plot of MR estimate assessed the relative intake of sugar on HER2 positive breast cancer risk; **(c)** Leave-one-out analysis of MR estimate assessed the relative intake of sugar on Luminal B breast cancer risk; **(d)** Leave-one-out analysis of MR estimate assessed the relative intake of sugar on HER2 positive breast cancer risk.
